# Chitosan–Glycolic Acid Gel Modification of Chloride Ion Transport in Mammalian Skin: An In Vitro Study

**DOI:** 10.3390/molecules28186581

**Published:** 2023-09-12

**Authors:** Olga Zavyalova, Dominika Dąbrowska-Wisłocka, Konrad Misiura, Iga Hołyńska-Iwan

**Affiliations:** 1 Department of Chemical Technology and Pharmaceuticals, Faculty of Pharmacy, Ludwik Rydygier Collegium Medicum in Bydgoszcz, Nicolaus Copernicus University in Torun, 87-100 Torun, Poland; zavolg@cm.umk.pl (O.Z.); dominika.dabrowska@cm.umk.pl (D.D.-W.);; 2 Department of Pathobiochemistry and Clinical Chemistry, Faculty of Pharmacy, Ludwik Rydygier Collegium Medicum in Bydgoszcz, Nicolaus Copernicus University in Torun, 87-100 Torun, Poland

**Keywords:** chitosan, epithelial sodium channels, chloride ion transport, cystic fibrosis transmembrane regulator, electric potential, electric resistance, skin

## Abstract

Chitosan, a polyaminosaccharide with high medical and cosmetic potential, can be combined with the beneficial properties of glycolic acid to form a gel that not only moisturizes the skin, but also has a regenerative effect. Its involvement in the activation of biochemical processes may be associated with the activity of skin ion channels. Therefore, the aim of the research was to evaluate the immediate (15 s) and long-term (24 h) effect of chitosan–glycolic acid gel (CGG) on the transepithelial electric potential and the transepithelial electric resistance (R) of skin specimens tested in vitro. Stimulation during immediate and prolonged application of CGG to skin specimens resulted in a significant decrease in the measured minimal transepithelial electric potential (PDmin). The absence of any change in the R after the CGG application indicates that it does not affect the skin transmission, or cause distortion, microdamage or changes in ion permeability. However, the reduction in potential may be due to the increased transport of chloride ions, and thus water, from outside the cell into the cell interior. Increased secretion of chloride ions is achieved by stimulating the action of the CFTR (cystic fibrosis transmembrane conductance). It can be assumed that chitosan gently stimulates the secretion of chlorides, while maintaining a tendency to reduce the transport of sodium ions, without causing deformation or tissue damage.

## 1. Introduction

**Figure 1:**
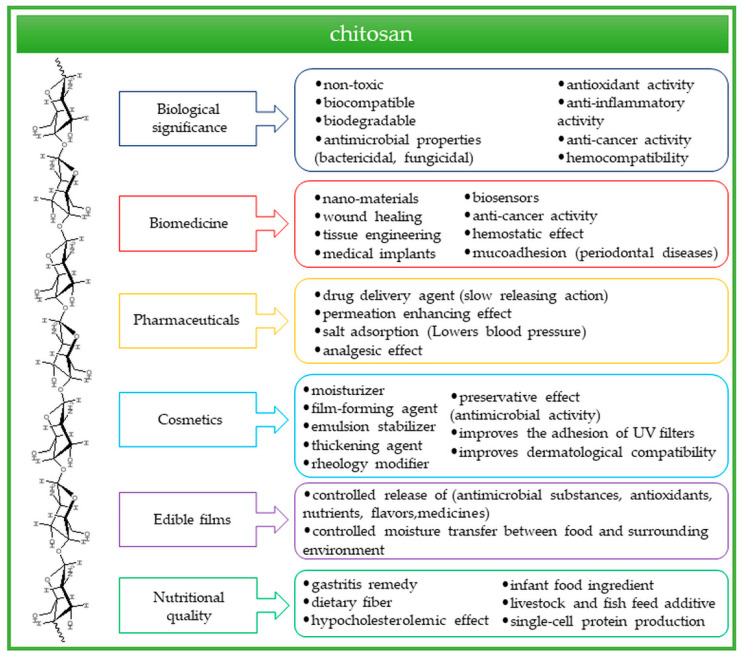
Properties and applications of chitosan.

Chitosan is a linear polyaminosaccharide obtained in the process of chemical or enzymatic deacetylation of chitin. It is a biologically derived raw material and the second most common polymer after cellulose. Chitosan is well-known for its applicability as a biocompatible, biodegradable and non-toxic material for biomedical, food-related, supplemental and cosmetic use [1,2,3,4]. The unique properties of chitosan result from the presence of amine and hydroxyl functional groups in its structure [3,4,5]. Its special features and biological activity, including antimicrobial, antioxidant, anti-cancer, anti-inflammatory, hemocompatible and hemostatic properties, also result from its chemical structure and make it an excellent raw material for biomedical applications [6,7] (Figure 1).

The antimicrobial effect is closely related to the presence of positively charged amino groups in the structure of the molecule, which interact with the negatively charged surface of the cell walls of microorganisms. This leads to damage to the membranes and destruction of the internal structures of the pathogens [8]. Chitosan also has a high chelating ability, supporting antimicrobial activity [5,6]. This makes chitosan a potential replacement for some antibiotics that are effective against drug-resistant bacteria [9]. Moreover, chitosan acts as an antioxidant that neutralizes free radicals by creating stable radicals with its functional groups [5].

The polymer, due to targeting pro-inflammatory cytokines and inhibiting their expression, exerts an anti-inflammatory effect [5]. In addition, it stimulates humoral and cellular immunity. It has been proven that chitosan inhibits the growth of cancer cells and is helpful in gene therapy aimed towards the direct delivery of genetic material [10]. The bioactivity of the polymer is conditioned by the degree of deacetylation and molecular weight [8].

The presence of amino and hydroxyl groups in the chitosan molecule make it easy to modify it with other bioactive compounds. Chemical modifications include cross-linking, graft copolymerization, carboxymethylation, etherification, esterification, O-acetylation, hydroxyalkylation, sulfonation, acetylation, quaternization, Schiff base reaction, grafting and several others. These modifications not only improve solubility and affect rheological properties, thermal stability and oxidation resistance, but they also impart new biological activity [4]. Chitosan modification products with desired properties are, in various forms, components of biomaterials—for example, hydrogels [11], nanofibers [12], microparticles [13], nanoparticles [14] or scaffolds [14,15,16].

Chitosan is of particular importance in skin regeneration processes. It has the ability to increase the influx of phagocytic cells to the site of infection and affects the proliferation of fibroblasts. It also stimulates the production of cytokines and activates macrophages and neutrophils, which cleanse wounds. This polymer is an inhibitor of metalloproteinase 2 (MMP-2), which is present in skin fibroblasts and a hydrolyzing type IV collagen. Inhibiting the action of MMP-2 enables the proper reconstruction of damaged tissues in the case of chronic wounds [17,18]. These properties make chitosan a great potential tool in skin tissue engineering [5,19,20].

**Figure 2:**
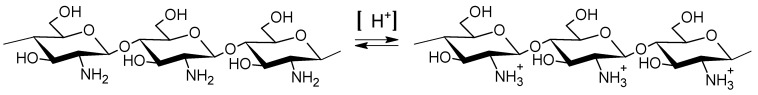
Scheme of chitosan protonation under acidic conditions.

The study of chitosan properties requires its prior dispersion in an aqueous environment, as is the case with most polysaccharides. Chitosan dissolves only in acidic solutions, at a pH value lower than pH6. This is due to the presence of intermolecular hydrogen bonds that prevent the dissolution of chitosan in water or organic solvents. As a result of protonation of amino groups in an acidic environment (Figure 2), chitosan forms a water-soluble cationic polyelectrolyte [3,21].

**Figure 3:**
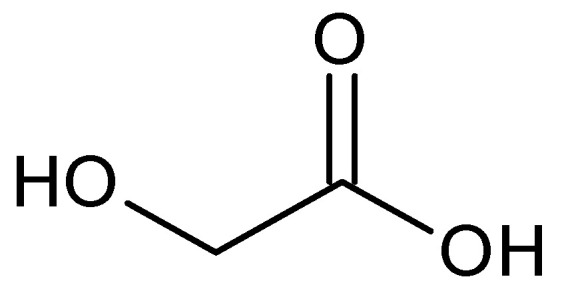
Chemical structure of glycolic acid.

In this study, glycolic acid was selected to lower the pH of the solution and form a water-soluble chitosan polycation. Glycolic acid (hydroxyacetic acid, Figure 3) is the simplest hydroxycarboxylic acid. It belongs to the group of alpha hydroxy acids, among which it has the smallest molecule [22].

Glycolic acid is present in many sugar-rich plants, including sugar cane, sugar beet, apples and grapes. And for years it has been well known among specialists in the cosmetics and dermatology community as a multifunctional skin care product [22,23]. The small size of the acid molecule determines its excellent penetration into the skin layers, intensifying the degradation of corneodesmosomes responsible for corneocyte adhesion. Due to this, glycolic acid is a popular exfoliant. The effectiveness of glycolic acid as a peeling exfoliant depends primarily on the amount that is in the most biologically active free acid form, its pH value and its concentration. As with all alpha hydroxy acids, glycolic acid may cause irritation and erythema of the skin, which is intensified at high acid concentration and lower pH [22].

Depending on the concentration used, glycolic acid can have an anti-inflammatory effect. At a concentration of 0.1 mM, it has a significant photoprotective effect on human keratinocytes by regulating the secretion of cytokines induced by UVB radiation and the secretion of chemokines in keratinocytes [24].

**Figure 4:**
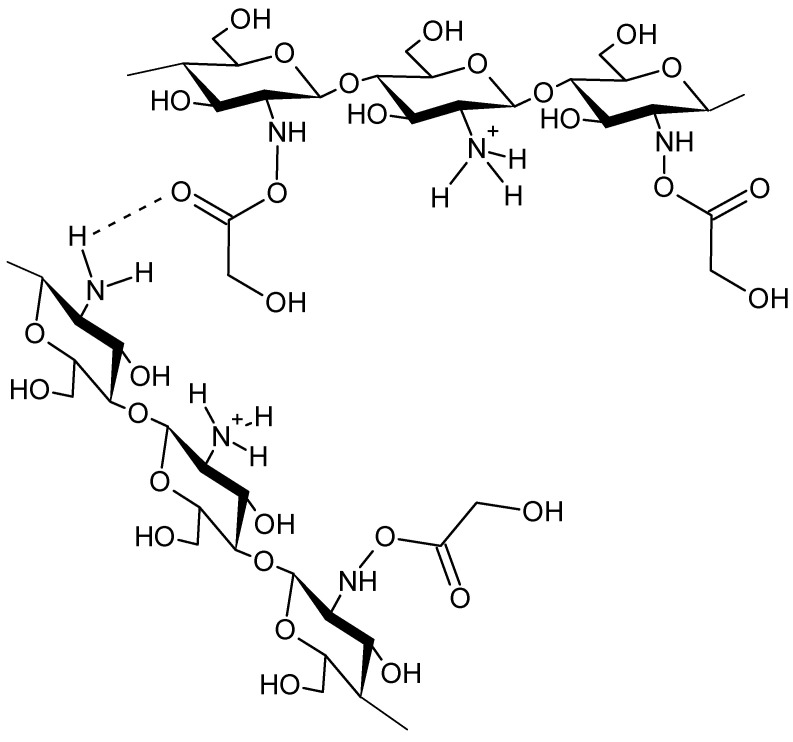
Scheme of chitosan–glycolic acid interaction.

CGG is formed as a result of interactions between the positively charged protonated amine groups of chitosan and the negative charges present in the glycolic acid molecule, with the formation of various types of bonds connecting different chitosan chains (Figure 4) [21]. The obtained gel can be used not only in various skin anti-aging agents, but also in wound dressing materials [4] and improved drug delivery systems [25].

Due to the high molecular weight of chitosan, it seems that only the superficial effect of water retention on the skin can be expected. However, changes in the skin under the influence of chitosan indicate the activation of more advanced physiological processes. Studies of changes in electrophysiological parameters of the skin, such as skin electrical potential or tissue resistance, can shed light on the nature of skin changes.

Mammals’ skin, due to the close intercellular connections and its multilayer, bipolar structure, is characterized by an electrophysiological parameter of electrical resistance and electric potential [26,27,28,29]. The multilayered skin structure, tight intercellular connections and the high content of keratin and lipids in cells determine high resistance values [26,29,30]. The value of electrical resistance also depends on the activity of sweat glands, the degree of skin hydration and the integrity of the epidermis, because damage may cause a decrease in electrical resistance [26,29,30,31]. Rabbit skin seems to be an appropriate experimental model to study the effect of various substances on the properties of tissue in relation to human skin. There are even reports that rabbit skin is more sensitive than that of humans [27,31,32,33]. The properties of the skin can be assessed by checking its integrity and the transport of ions through the membranes of its building cells [30,34]. This can be done by examining the changes in the electric potential of tissues exposed to various factors [26,27,31,32,35]. The transport of skin ions is essential for the proper functioning of the entire organism [36,37]. The functioning of epithelial sodium channels (ENaCs) in keratinocytes is related to the transport of water through the skin layer, the action of immunocompetent cells and wound healing. The transport of chloride ions is via the transmembrane regulator of cystic fibrosis (CFTR) and the chloride channel (CLCA) [38,39]. The activity of chloride channels seems to be important for water flow and for dehydration and overhydration of the skin microenvironment [37,40]. CFTR channels act on cellular regulators that influence, for example, the functioning of ENaCs [37,38]. Changes in the functioning of sodium and/or chloride channels may underlie problems with hypersensitivity to pain [41,42], the onset of hypersensitivity and/or allergy [41,43], issues with regeneration and healing [40] and atopic dermatitis [39].

The aim of the study was to evaluate the immediate (15 s) and long-term (24 h) effects of chitosan gel with glycolic acid on both the transepithelial electric potential and the transepithelial electric resistance of the skin specimens tested in vitro. The transepithelial electric potential reflects changes in transepithelial ion transport pathways following the use of chitosan gel with glycolic acid. Currently, there are no direct scientific reports on the effects of chitosan on electrophysiological parameters of the skin, which indicate changes in the functioning of ion channels.

## 2. Results

Transepithelial electrical resistance was measured initially before and after mechanical and mechanical–chemical stimulations for skin fragments treated with both iso-osmotic Ringer’s solution and chitosan. Results ranged from 2474 Ω*cm^2^ (median, R final chitosan incubation) to 3450 Ω*cm^2^ (median, R final RS incubation). There were no statistically significant differences in any of the study groups (Table 1, Wilcoxon test) or between the groups (Table 1, Mann–Whitney test). The resistance of the skin specimens did not change under the influence of chitosan.

The initial PD, measured without stimulation for the skin fragments held in the iso-osmotic RS, was −0.53 mV (median). However, the final one was statistically significantly reduced (Table 1, Wilcoxon test) to the level of −0.86 mV (median). The initial PD measured for the chitosan treated skin specimens was −1.11 mV (median). The final PD measured after a series of mechanical and mechanical–chemical stimulations tended to increase to −0.53 mV (median), but this was not a statistically significant increase. Comparing the initial PD value measured for the control tissues and those treated with chitosan, a statistically significant reduction was demonstrated (Table 1, Mann–Whitney test). Meanwhile, the final PD was similar in both tissue groups.

The immediate effects of chitosan included a statistically significant decrease in measured PDmin to a value of −0.57 mV (median), compared to −0.4 mV (median) for the samples that underwent RS stimulation (Table 2, Wilcoxon test, RS incubation). Long-term retention of tissues in the chitosan solution and stimulation with the chitosan and Ringer’s solution resulted in a significant decrease of measured PDmin to the value of −1.31 mV (median) for chitosan stimulation and −1.06 mV (median) for RS stimulation. The PDmin values measured for these stimulations were similar (Table 2, Wilcoxon test, CGG incubation). The comparison of the two incubation conditions showed a significant decrease in the PDmin measured after stimulation with RS and with chitosan solution, performed under long-term stationary conditions (Table 3, Mann–Whitney test).

The PDmax measured under the conditions of incubation in Ringer’s solution and during stimulation with this solution, before chitosan administration, was 0.37 mV (median) positive, while during chitosan stimulation it decreased to 0.23 mV (median). These values did not differ significantly (Table 2, Wilcoxon test, RS incubation). During incubation in chitosan solution and stimulation with its solution and with RS, the PDmax was similar and amounted to 0 mV (median). Comparison of the two incubation conditions showed no significant change in PDmax for RS stimulation (Table 3, Mann–Whitney test). The analysis of PDmax values measured for stimulation with chitosan solution showed a statistically significant reduction of this parameter (Table 3, Mann–Whitney test).

The administration of sodium (A) and chloride (B) ion transport blockers caused changes in PDmax and PDmin in a similar manner, regardless of whether chitosan was administered for 15 s during the stimulation or whether incubation for 24 h was used (Table 4).

The applied series of stimulations caused reproducible changes in the measured PDmin and PDmax potential, regardless of the applied incubation conditions and the type of stimulation (Table 2 and Table 4). PDmin and PDmax values measured during stimulation were statistically significantly different from PD measured under stationary conditions, i.e., without stimulation (Table 5, Wilcoxon test).

## 3. Discussion

In this study, the influence of chitosan–glycolic acid gel on the electrophysiological parameters, such as electric potential and resistance of tissues of the skin, has been investigated.

The positive effects of chitosan application have been confirmed in many scientific studies. Chitosan is beneficial for the skin, hair and nails [11,12,13,14,15,16,44,45]. Despite its positive effects on the skin, chitosan is not absorbed by it. It creates a hydrophilic film on the skin surface, which effectively reduces transepidermal water loss from the epidermis and supports the renewal of the skin’s natural hydro-lipid coat [44,45]. Chitosan has antimicrobial and antioxidant properties, supports skin regeneration processes, and has anti-inflammatory and anti-cancer effects.

The advantageous effect of glycolic acid has been confirmed in relation to various skin problems connected to keratinization disorders. Glycolic acid peeling treatments affect the lightening of melasma and post-inflammatory hyperpigmentation, are an element of anti-acne therapy in adolescents and adults and reduce actinic or seborrheic keratosis [46,47,48]. Glycolic acid also improves the penetration of other exfoliants. The anti-aging effect of glycolic acid is related to its ability to stimulate the production of hyaluronic acid and collagen [49].

The use of glycolic acid creates an acidic environment that allows chitosan to be introduced into the solution. Glycolic acid, having the smallest molecule among hydroxy acids, is a weak acid (pKa = 3.83). The chitosan chain will interact not only with the hydrogen ions formed during acid dissociation, but also with counter-anions (glycolate), which contributes to better dispersal of the chitosan [50,51,52].

It is difficult to explain the beneficial influence of chitosan on the skin only on the basis of its superficial effects. It can be assumed that chitosan is involved in the activation of biochemical processes responsible for the functioning of the skin, including the activity of ion channels. However, there are no direct studies regarding the effect of chitosan on such processes.

Changes in ion transport in the skin can be measured using a modified Ussing chamber [31,32]. The modification consists of placing the tissue in a horizontal position and applying a stimulus. Stimulation is based on the free release of fluid onto the surface of the examined tissue. Full-thickness skin fragments with preserved vitality, layering and physiological intercellular spaces, as well as active nerve endings and functioning channels and ion pumps, are examined. The assessment of ion transport is important for the evaluation of processes such as tissue hydration; the ability to receive stimuli; and the function of immunocompetent cells and melanocytes [31,32]. So far, the influence of chitosan and glycolic acid on the electrophysiological parameters of the skin has not been studied. The effect of CGG on the transport of sodium ions, chlorides and water in a multilayer structure seems to be extremely important for inferring the moisturizing, immunogenic effect or the maintenance of a uniform skin tone.

Our studies were carried out in stationary conditions (RS, CGG), during which mechanical and/or mechanical–chemical (A, B, AB) stimulation caused changes in the ion transport.

The lack of changes in the electrical resistance after the use of chitosan proves that the incubation of the skin specimens in the applied concentration of chitosan solution does not change the skin permeability (Table 1, Mann–Whitney). It also does not cause deformations, microdamage or changes in the ion permeability. The resistance values are stable and the tissue was alive and reactive throughout the experiment (Table 2, Wilcoxon, control and CGG). All skin specimens used were alive and retained full structure and compactness as well as active nerve fibers. Chitosan gel with glycolic acid applied to the outer skin layer did not change the vitality and compactness of the tissues. It also did not increase the ion and water permeability of the analyzed fragments. The ability to respond to the applied mechanical and/or mechanical–chemical stimulus was not affected by CGG, as tissues treated with CGG reacted analogously to control fragments (Table 5, Wilcoxon test).

Maintaining a negative charge on the surface of skin cells depends on the proper transport of chloride ions to the surface of the skin and the penetration of sodium ions into the cells [27,31,32]. The use of ion transport inhibitors amiloride and bumetanide allowed the inhibition of the entire pathway for individual ions and obtaining layers, cells that cannot secrete chloride or absorb sodium [1,2,27,31,32]. A reduction of the potential measured in stationary conditions (PDinitial) was demonstrated in comparison with preparations treated with Ringer’s solution and with CGG (Table 1, Mann–Whitney, PDinitial). The reduction of the potential may result from the increased transport of chloride ions from the cell, and thus cause an influx of water in the intracellular direction [1,2]. After a series of stimulations, the potential value (PDfinal) does not differ from the control. The cells transported available chloride ions and water, so there is no room for excessive ion/water influx and cell swelling (Table 1, Mann–Whitney, PDfinal). Chitosan and glycolic acid gel does not increase the activity of the sodium–potassium pump maintaining the difference in potential under stationary conditions. The physiological activity is preserved (Table 5, Wilcoxon, CGG). The constantly occurring, ion transport enabling reaction to external and internal stimuli was not affected by the CGG solution. The generation of a more electronegative PD after incubation in CGG is most likely related to the intensification of chloride secretion to the surface. It is not possible to absorb such a large amount of sodium ions without interfering with the cell volume, which was ruled out after examining R, which was not changed after the application of CGG.

The absence of changes in the transepithelial transport of sodium ions after the use of CGG, both during stimulation and in case of prolonged action, is important for maintaining skin tissue homeostasis [31,33,36,37,43,53]. Each time, the intensification of the transport of sodium ions is associated with the movement of water towards the outer layers and its loss [1,31,37]. Additionally, the displacement of sodium ions is associated with the response to sensory stimuli [42] and the response to the triggers of local inflammation [43,53]. Substances changing the sodium transport in the skin may cause hypersensitivity reactions, pain and hyperreactivity [32]. The applied CGG solution did not cause such reactions under the proposed experimental conditions (Table 1, Table 2 and Table 4).

Increased secretion of chloride ions is achieved thanks to the activation of the CFTR transporter (cystic fibrosis transmembrane conductance). This channel also acts out the role of a cell volume regulator, by inhibiting the ENaC channel (epithelial Na channel) and stimulating other chloride ion transport channels present in the apical membrane of skin cells [1,27,31,32]. The stimulation of the CFTR channel is also associated with the inhibition of the 2Cl-K-Na cotransporter [54]. In addition, CFTR stimulation is associated with the maintenance of the physiological pH of cells in the cell cytoplasm due to the interaction with the sodium–potassium pump and potassium channels [55]. Access to magnesium ions is essential for the activation and regulatory action of CFTR [27]. The CFTR channel present in the sweat channels is inhibited by bumetanide [1,2,26]. It can be assumed that the applied chitosan solution gently stimulates CFTR to chloride secretion, maintaining a tendency to decrease sodium ion transport (changes in potential—PD, Table 3 and Table 4) and to maintain a constant cell volume. At the same time, it causes a slight increase in the amount of water in the intracellular spaces, without causing deformations or tissue damage (no change in R, Table 1). This confirms the data [21,23,24,45] that chitosan and glycolic acid have a moisturizing effect on the skin tissue, allowing the preservation and/or replenishment of water in the cells. The thesis about the effect of chitosan on ion channels is confirmed by reports on the effectiveness of the use of chitosan-based nanosystems in order to modify the operation of ion channels, e.g., reducing ENaC activity [51] and stimulating CFTR activity [52].

## 4. Materials and Methods

### 4.1. Animals

Specimens were excised from adult albino New Zealand rabbits of both sexes, weighing 3.5 to 4.0 kg and ranging in age from three to four months. Animals were subjected to asphyxiation with CO_2_. The gas causes respiratory depression, reduces the contractility of the heart muscle and has a beneficial effect on the neuromuscular system. The death of each animal was confirmed by two methods, by a qualified person. The obtained preparations, isolated from the skin of ears, were stripped of hair and cleaned. The experiment consisted in examining tissues taken from sacrificed animals. To maximize the use of sacrificed animals, each rabbit’s trachea, intestines, skin, liver, kidneys and muscles were also collected for other experimental procedures. The isolated tissues were submerged and incubated in the appropriate solution according to the experimental protocol. Rabbit skin prepared this way contains corneocytes, keratinocytes (95%), fibroblasts, immunocompetent cells, hair follicles, and nerve fiber endings.

### 4.2. Experimental Procedure

A modified Ussing chamber was used in the experiments. The tissue was mounted in a horizontal position, which allowed the stimulus to be applied to the examined surface [12]. The nozzle outlet was located at a safe distance from the tissue structure (approx. 5–7 mm). The surface of the external side of the tissue, which was gently rinsed with the solution, was 1 cm^2^. The fluid flowing through the nozzle moved perpendicularly to it. A single stimulation lasted 15 s and the fluid was administered in a volume of 1 mL (0.06 mL/s), during which the analyzer recorded noticeable changes in the transepithelial electric potential. The modified Ussing chamber used consisted of two parts, and the tissue placed between the half-chambers acted as a partition. In order to equalize the pressure after administration of the stimulation fluid, went-holes had been placed in the upper half-chamber to allow the removal of excess solution.

Mechanical stimulation was performed using a Ringer’s solution (RS), a stimulating fluid without a chemical component that only mechanically affected the isolated tissue. The combined mechanical–chemical stimulation was achieved through the use of a stimulus, CGG and/or amiloride and/or bumetanide. The immediate effect was tested with a 15 s mechanical–chemical stimulation by chitosan solution of the skin specimens. The prolonged effect was tested after applying the chitosan solution for 24 h to the external surface of the skin taken from the ear of a rabbit.

Tissues were kept in the dark at constant temperature (25 °C) and humidity (55%). After that time, measurements of electrophysiological parameters were performed:

(1) In stationary conditions: transepithelial electric potential (PD, mV) continuously measured, transepithelial electric resistance (R, Ω*cm^2^) measured after stimulation and counted according to Ohm’s law.
(2) During 15 s stimulation: minimal transepithelial electric potential (PDmin), maximal transepithelial electric potential (PDmax).

Measurement of electrophysiological parameters lasted 30 min for each tissue specimen. The following parameters were measured during the experiment:

- R—transepithelial electrical resistance recorded while the tissue sample was exposed to a current with a stimulus intensity of ±10 μA; then, after measuring the voltage change, calculations were made according to the Ohm’s law (Ω*cm^2^).
- PD—changes in transepithelial electric potential difference measured in stationary conditions, i.e., without stimulation, recorded continuously (mV).
- PDmax and PDmin—minimal and maximal transepithelial electric potential difference measured during a 15 s stimulation (mV).

### 4.3. Chemicals

For the experiment the following solutions were used:

- Ringer’s solution (RS)—(K^+^ 4.0 mM; Na^+^ 147.2 mM; Ca^2+^ 2.2 mM; Mg^2+^ 2.6 mM; Cl^−^ 160.8 mM; Hepes 10.0 mM), solution with iso-osmotic properties.
- Mineral compounds (NaCl, CaCl_2_, KCl, MgCl_2_) were purchased in Avantor Performance Materials Poland S.A., Poland.
- Chitosan–glycolic acid gel in RS (CGG) was prepared by dissolving chitosan (2.6% *w*/*v*) in 30 mL of aqueous glycolic acid solutions and put away for 24 h in a dark place. After this time, it was diluted with 500 mL of the Ringer’s solution. Chitosan and glycolic acid were obtained from ACROS Organics, Poland, and used without further purification.
- A—amiloride, 3,5-diamino-6-chloro-2-carboxylic acid, 0.1 mM, (Sigma-Aldrich, St. Louis, MO, USA), inhibitor of ENaCs, used as an inhibitor of transepithelial sodium transport pathways.
- B—bumetanide, 3-butylamino-4-phenoxy-5-sulfamoylbenzoic acid, 0.1 mM (Sigma-Aldrich, St. Louis, MO, USA), inhibitor of Na-K-Cl cotransporter, used as an inhibitor of transepithelial chloride transport pathways.
- AB—a solution of amiloride (A, 0.1 mM) and bumetanide (B, 0.1 mM).

### 4.4. Data Analysis

The Ussing chamber was connected to the EVC 4000 measuring instrument (WPI, Worcester, MA, USA) coupled with the MP150 (Biopac, Goleta, CA, USA), a computer system which enables the recording of experimental results. The non-parametricity of the data distribution was confirmed by the Kolmogorov–Smirnov test, with the Lilliefors correction.

The results were presented as median and lower and upper quartile. Due to the fact that incorrect distribution of data was demonstrated, non-parametric tests, Wilcoxon test and U Mann–Whitney test with significance level *p* < 0.005 were used for the analysis. The obtained values were statistically analyzed using the Statistica 13.1. Programs.

## 5. Conclusions

The influence of chitosan–glycolic acid gel on the electrophysiological parameters, such as electric potential and resistance of skin tissues, was investigated. The studies were carried out in stationary conditions (RS, CGG) during which mechanical and/or mechanical–chemical (A, B, AB) stimulations caused changes in ion transport. A modified Ussing chamber was used in the experiments.

Studies have shown that stable values of electrical resistance are observed after the use of chitosan. This means that incubation in the applied concentration of chitosan solution does not increase the skin’s permeability, the tissue remains alive and reactive. The observed decrease in stationary potential (PDinitial) probably indicates an increased transport of chloride ions, and thus water, from outside the cell into the cell interior. After the start of the stimulations, the potential value (PDfinal) did not differ from the control, the cells transported the available chloride ions and water and there was no excess ion/water flux and no cell swelling.

Chitosan maintains the potential difference under stationary conditions, but does not increase the activity of the sodium–potassium pump. Physiological activity is maintained, which is important for maintaining skin tissue homeostasis. Increased secretion of chloride ions is initiated by stimulating the action of CFTR. This causes a slight increase in the amount of water in the intercellular spaces without causing distortions and damage to the tissue.

## Figures and Tables

**Table 1:** Transepithelial resistance (R) and transepithelial electric potential (PD) measured under steady-state conditions with immediate and prolonged effects of chitosan–glycolic acid gel solution on skin specimens.

		PD Initial (mV)	PD Final (mV)	Wilcoxon Test (*p*)	R Initial (Ω∗cm^2^)	R Final (Ω∗cm^2^)	Wilcoxon Test (*p*)
Incubation: RS (n = 40)	median	−0.53	−0.86	0.000103	2721	3450	0.667697
	lower quartile	−0.24	−1.31		1422	1467	
	upper quartile	0.23	−0.15		4855	5024	
Incubation: CGG (n = 32)	median	−1.11	−0.53	0.099541	2817	2474	0.379375
	lower quartile	−1.48	−0.86		1050	1013	
	upper quartile	−0.09	0		5916	5203	
Mann–Whitney test (*p*)	RS vs. Chitosan	0.002873	0.219698		0.66735	0.62078	

Abbreviations: RS—iso-osmotic Ringer solution, CGG—chitosan–glycolic acid gel in RS, PD—transepithelial potential difference of epithelial skin surface measured in stationary conditions (mV), R—resistance measured in stationary conditions (Ω∗cm^2^), *p* < 0.05.

**Table 2:** Minimal (PDmin) and maximal (PDmax) transepithelial electric potential, measured during 15 s mechanical (RS) and mechanical–chemical (chitosan–glycolic acid gel) stimulations of skin specimens treated with immediate and prolonged exposure to chitosan–glycolic acid gel solution.

Incubation: RS (n = 40)				Incubation: CGG (n = 32)			
Stimulation		PDmin (mV)	PDmax (mV)	Stimulation		PDmin (mV)	PDmax (mV)
RS	median	−0.4	0.37	CGG	Median	−1.31	0
	lower quartile	−0.98	−0.23		lower quartile	−2.06	−0.97
	upper quartile	0	0.97		upper quartile	−0.36	0.58
CGG	median	−0.57	0.23	RS	Median	−1.06	0
	lower quartile	−1.16	−0.34		lower quartile	−2.04	−0.26
	upper quartile	−0.15	0.69		upper quartile	−0.27	0.45
	Wilcoxon test (*p*)	0.014024	0.112656		Wilcoxon test (*p*)	0.378920	0.243286

Abbreviations: RS—iso-osmotic Ringer solution, CGG—chitosan–glycolic acid gel in RS, PDmin—minimal transepithelial potential difference during 15 s stimulation of epithelial skin surface (mV), PDmax—maximal transepithelial potential difference during 15 s stimulation of epithelial skin surface (mV), *p* < 0.05.

**Table 3:** Mann–Whitney test results for minimal (PDmin) and maximal (PDmax) transepithelial electric potential, measured during 15 s mechanical (RS) and mechanical–chemical (chitosan–glycolic acid gel) stimulations of skin specimens exposed to chitosan–glycolic acid gel solution immediately and for a prolonged period.

PDmin	*p*
RS stimulation in RS incubation vs. RS stimulation in CGG incubation	0.000502
CGG stimulation in RS incubation vs. CGG stimulation in CGG incubation	0.002292
PDmax	*P*
RS stimulation in RS incubation vs. RS stimulation in CGG incubation	0.090533
CGG stimulation in RS incubation vs. CGG stimulation in CGG incubation	0.043357

Abbreviations: RS—iso-osmotic Ringer solution, CGG—chitosan–glycolic acid gel in RS, PDmin—minimal transepithelial potential difference during 15 s stimulation of epithelial skin surface (mV), PDmax—maximal transepithelial potential difference during 15 s stimulation of epithelial skin surface (mV), *p* < 0.05.

**Table 4:** Minimal (PDmin) and maximal (PDmax) transepithelial electric potential measured during 15 s mechanical–chemical stimulations of skin specimens treated with immediate and prolonged exposure to chitosan–glycolic acid gel solution.

Incubation		RS (n = 40)		Chitosan (n = 32)	
Stimulation		PDmin (mV)	PDmax (mV)	PDmin (mV)	PDmax (mV)
B	Median	−0.64	0.08	−0.63	0.36
	lower quartile	−1.45	−0.43	−1.28	−0.06
	upper quartile	−0.2	0.67	−0.19	1.94
A	Median	−0.86	−0.08	−0.96	0.06
	lower quartile	−1.28	−0.72	−3.03	0
	upper quartile	−0.21	0.69	−0.26	0.98
AB	Median	−0.54	0.23	−0.54	0.12
	lower quartile	−0.74	−0.57	−1.16	−0.59
	upper quartile	0.07	0.92	−0.12	2.12

Abbreviations: RS—iso-osmotic Ringer solution, CGG—chitosan–glycolic acid gel in RS, A—amiloride (0.1 mM) solution; B—bumetanide (0.1 mM) solution; AB—mixture A (0.1 mM) and B (0.1 mM) solutions; PDmin—minimal transepithelial potential difference during 15 s stimulation of epithelial skin surface (mV); PDmax—maximal transepithelial potential difference during 15 s stimulation of epithelial skin surface (mV).

**Table 5:** Results of Wilcoxon test for minimal (PDmin) and maximal (PDmax) transepithelial electric potential measured during 15 s mechanical (Ringer solution) and mechanical–chemical (chitosan solution) stimulation of skin specimens treated with immediate and prolonged exposure to chitosan glycolic acid gel solution.

Incubation: RS (n = 40)	*p*
PD vs. PDmin for RS stimulation	<0.001
PD vs. PDmax for RS stimulation	<0.001
PD vs. PDmin for CGG stimulation	<0.001
PD vs. PDmax for CGG stimulation	<0.001
Incubation: CGG (n = 32)	*p*
PD vs. PDmin for CGG stimulation	<0.001
PD vs. PDmax for CGG stimulation	<0.001
PD vs. PDmin for RS stimulation	<0.001
PD vs. PDmax for RS stimulation	<0.001

Abbreviations: RS—iso-osmotic Ringer solution, CGG—chitosan–glycolic acid gel in RS, PDmin—minimal transepithelial potential difference during 15 s stimulation of epithelial skin surface (mV), PDmax—maximal transepithelial potential difference during 15 s stimulation of epithelial skin surface (mV), *p* < 0.05.

## Data Availability

Data will be able on request, email: igaholynska@cm.umk.pl.

## Sample Availability

Samples of the compounds are available from the authors.
